# “The Real Thing I Struggle with is Other People’s Perceptions”: The Experiences of Autistic Performing Arts Professionals and Attitudes of Performing Arts Employers in the UK

**DOI:** 10.1007/s10803-020-04517-0

**Published:** 2020-04-30

**Authors:** Eleanor Buckley, Elizabeth Pellicano, Anna Remington

**Affiliations:** 1 UCL Centre for Research in Autism and Education (CRAE), University College London, London, WC1H 0NU UK; 2 Macquarie School of Education, Macquarie University, Sydney, Australia

**Keywords:** Autism, Employment, Employers, Support, Disclosure, Arts

## Abstract

This research examined in-depth the employment experiences of autistic performing arts professionals and the attitudes and adjustments of performing arts employers. We interviewed 18 autistic performing arts professionals and 19 performing arts employers. Autistic performing arts professionals described facing challenges in the workplace. Some autistic professionals had access to support, but the majority felt that there was not enough available and highlighted many ways in which they could be better supported. Performing arts employers varied in their experiences of working with autistic people, many had limited knowledge about autism-specific support or relied on other professionals to provide it. These findings shed light on current unmet support needs of autistic performing arts professionals, and provide key recommendations for research and practice.

For many autistic adults, finding and maintaining work is a desired goal, but the current rates of employment in the United Kingdom (UK) for this group are low, with 16% of autistic adults being in full-time employment and 32% being in any kind of employment (National Autistic Society 2016). Although prevailing stereotypes suggest that autistic people prefer solitary, computer-based jobs, we know, at least anecdotally, that autistic people are employed in many different areas, including creative disciplines like the performing arts. The National Autistic Society’s employment survey (2016) reported that 11% of their 2080 autistic respondents stated that they hope to work in the arts or pursue acting careers. Our previous research has also demonstrated that a significant number of autistic people are pursuing careers in the performing arts (Buckley et al. 2020). Yet, there is currently no research on the experiences of autistic people currently employed in this specific field. This study sought to address this gap, by investigating the views and experiences of autistic performing arts professionals *and* performing arts employers in the UK.

In broader autism employment research, there is consensus that many autistic adults face often-substantial challenges in the workplace, mostly related to interactions with, or attitudes of, employers (e.g., Baldwin et al. 2014; Hurlbutt and Chalmers 2004; López and Keenan 2014; Unger 2002). Knowledge and attitudes of neurotypical employees (or employers) towards autistic colleagues have been highlighted as critical factors to the successful employment of autistic workers (Annabi and Locke 2019). These factors form part of the adapted Organizational Interventions Mitigating Individual Barriers (OIMIB) framework, originally created by Annabi and Lebovitz (2018), to address barriers that women face in the Information Technology (IT) industry. Annabi and Locke (2019) applied the OIMIB theoretical framework to interpret autism employment research and its impact in the IT industry and beyond, by focusing on individual, intervention, and organizational levels and how these interrelate to barriers faced by autistic people in employment.

Following the adapted OIMIB framework (Annabi and Locke 2019), a lack of employer knowledge about autism and how autistic people can be supported in the workplace may result in them not receiving the necessary workplace support. Employers play a crucial role in how accessible workplaces are for those with disabilities (Unger 2002), but many employers do not understand the most effective ways of working with disabled people (Rashid et al. 2017). Furthermore, while employers often express favourable attitudes towards workers with disabilities when asked (Kregel and Tomiyasu 1994), this is not always reflected in their hiring practices (Copeland 2007; Unger 2002). For employers who do not currently work with disabled employees, this lack of experience can result in negative beliefs around whether potential employees would have the necessary knowledge, skills and abilities to perform needed jobs, alongside fears concerning the cost of necessary accommodations and negative customer reactions (Fraser et al. 2010; Graffam et al. 2002; Lengnick-Hall et al. 2008). These attitudes and beliefs are often based on stereotypes rather than experiences, and may be a significant barrier to employment for disabled—including autistic—people (Ju et al. 2013).

Even when employers are willing to work with autistic employees, research has shown that employers lack confidence providing appropriate workplace support without the guidance of disability employment organizations or other external support (Howlin et al. 2005; Remington and Pellicano 2018; Scott et al. 2015). Autistic employees can require on-going support such as a structured and task-adapted environment (Scott et al. 2018) and a working environment that does not trigger sensory hypersensitivities, such as aversions to particular lighting and/or sounds (Marco et al. 2011). Yet employers often place the onus of responsibility on the autistic employee, rather than the employer, to make these kinds of adjustments in order to maintain employment and meet any productivity requirements (Scott et al. 2018).

## The Current Study

The adapted OIMIB framework (Annabi and Locke 2019) claims that autistic employees will experience fewer barriers when neurotypical colleagues are knowledgeable about autism and when they have positive attitudes towards autism. The existing research supports these claims: the majority of workplaces and employers do not appear currently to have adequate levels of awareness or support available to autistic employees, and our previous work established that there are autistic people working in the performing arts with unmet support needs (Buckley et al. 2020).

The current study extended this work to the field of the performing arts in the UK by eliciting the views of autistic performing arts professionals *and* performing arts employers. Our specific aims were twofold. First, we sought to understand the views of autistic performing arts professionals and their experiences of working with neurotypical colleagues and employers. We also examined the extent and nature of any occupation-based support they received in their workplaces and whether they perceived any such support to meet their needs. Second, we also spoke with performing arts employers to understand their attitudes and levels of knowledge about autism, how confident they were about working with autistic people, and whether they knew how best to support them in the workplace.

## Method

### Participants

In total, 37 participants took part in this study: 18 autistic performing arts professionals (7 female, 9 male, 2 non-binary or other) and 19 performing arts employers (10 female, 9 male). There was a slightly higher proportion of autistic professionals who reported ethnicities that were non-white, were non-binary, or had an intellectual disability than UK population prevalence estimates (Government Equalities Office 2018; UK census figures: Office for National Statistics 2011). The performing arts employers were predominantly white and none had intellectual disability. Participants were recruited through convenience sampling methods, purposive targeting of autistic performing arts professionals and performing arts employers through the Royal Academy of Dramatic Art and promotion on social media. Demographic information can be found in Table 1.

**Table 1:** Participant characteristics

	Autistic performing arts professionals N = 18	Performing arts employers N = 19
Age		
Mean (SD, years)	32.6 (12.1)	44 (9.3)
Median (years)	28.5	43.5
Range (years)	19–61	31–58
Ethnicity		
White	15	18
Black	2	
Asian		1
Other	1	
Gender		
Female	7	10
Male	9	9
Non-binary or other	2	
Intellectual disability	3	

The autistic professionals had been working in the performing arts for varying lengths of time, ranging from under 1 year to over 20 years (median = 6). The majority interviewed were primarily working as performers, while some worked as production technicians. Most reported working in more than one type of performing arts role over the course of their careers, such as being both a performer and a member of production staff, or as a performer and a writer or theatre maker. They were UK-based at the time of interview, although some had worked both in the UK and abroad. All self-identified as autistic, with 17 reporting having received an independent clinical diagnosis of an autism spectrum condition according to DSM-IV or DSM-5 criteria (American Psychiatric Association 1994, 2013).^1^

The performing arts employers had varied roles within the performing arts: there were directors, casting directors, artistic/creative directors, agents, and heads of production, technical, diversity, and access. A minority (n = 3) specifically worked with disabled performers or had roles that focused on supporting disabled performing arts employees. Systematic data were not collected on how long employers had worked within the performing arts, although many discussed careers that had spanned over 20 years. The employers were UK-based, although some had also worked abroad over the course of their careers.

### Measures

Semi-structured interviews were conducted with all participants. Interviews were recorded with participants’ prior consent and professionally transcribed verbatim. In the interviews with autistic professionals, participants were asked open-ended questions about their likes and dislikes concerning their workplaces, and if they had ever asked for, needed, or would like support in relation to their working environments or careers. In the interviews with employers, participants were asked about their current knowledge of autism, whether they had any experience working with autistic people, and if they knew how or where to find support for either an autistic employee or themselves if needed. While some employers explicitly stated that they had worked with autistic people, many reported being unsure. All employers were asked to reflect on their experiences working with people that they had both explicitly known or suspected to be autistic, and also asked to consider how they might go about potentially working with autistic people in the future.

### Data Analysis

Participants’ open-ended responses were analysed using reflexive thematic analysis, as detailed by Braun and Clarke (2006, 2019). The transcripts were analysed from an inductive (bottom-up) perspective where themes were created within a ‘contextualist’ method of critical realism (Willig 1999). The first and last authors carried out the thematic analysis and approached the analysis from the perspectives of psychology researchers who do not identify as autistic, and therefore analysed the data from the perspective of outside interpreters.

Data were initially coded separately by group (autistic professionals, employers) with focus on the semantic content of the data but, after considerable discussion, the authors agreed that many codes were shared across the two groups and so we decided to look at all of the data together, re-coding where necessary. The analysis was reflexive, so the authors moved backwards and forwards between the data and analysis. The authors met together several times to discuss the themes and subthemes, ensuring that the themes and their definitions encompassed the patterns of shared meanings across the entire data set and to resolve any inconsistencies.

### Procedure

Ethics approval was obtained from UCL Research Ethics Committee. All participants provided written informed consent prior to participating in this study. Participants completed individual semi-structured interviews over the phone, on Skype, or in-person, either on University premises or in a location of their choosing. Interviews with autistic professionals ranged in length from 16 to 54 min (M = 36 min), and for employers 11 to 42 min (M = 23 min). To preserve anonymity of the participants involved all quotations are identified only by letters.

## Results

We identified four themes. All themes and subthemes (italicised in the text), alongside example quotations, are listed in Table 2.

**Table 2:** Themes for autistic performing arts professionals and performing arts employers

Themes	Subthemes	Example quotes
*Autism can bring strengths*	Scholars of human expression	“I had to learn to understand body language, behaviours, facial expressions that sort of thing, I had to study them and from doing that, I think it’s made me in to a better actor.” Pro R
		“I just sort of … really developed this catalogue, this encyclopaedia of facial expressions and body language.” Pro K
	A detail-oriented approach	“I possibly just generally pay a bit more attention to the detail than other people might.” Pro L
		“There are characteristics of autism that seem really great for this kind of work, which is the attention to detail, and the determination to get things exactly right which is brilliant.” Emp M
	High engagement with the work	“Once I get into a project I can just sit down and I will do it all day.” Pro K
		“Hyper-focus is a bit beneficial for that, so I get really involved and I am like – yes so long hours don’t particularly bother me.” Pro J
	Seeing the world differently	“I think Asperger’s lends itself to creative thinking, that you see the world a bit differently, and that is actually quite a useful talent for the arts!” Pro J
		“They’re really creative.” Emp C
		“Autistic people in acting, there’s a boldness in trying things.” Emp A
*A challenging profession*	The workplace can be overwhelming	“I will be the person that’s likely to meltdown and loses it because I can’t hold it in, or I get too stressed.” Pro I
		“Walking into a new space she’s never been to, she gets real sensory overload.” Emp B
		“The lighting in some parts of the building can be a real barrier to autistic people. Sometimes, unfortunately, that’s the only space that we can do certain things.” Emp F
	Auditions can cause extreme anxiety	“Auditions I hate. I do not do well with auditions.” Pro O
		“Massive anxiety around auditions. And I do mean massive anxiety!” Pro I
	Struggle adapting to last minute changes	“Things do change last minute, and I find that really difficult, I find that really stressful internally.” Pro K
		“I understand that last minute changes to arrangements, which I am afraid does happen in this industry, can actually cause a bit of disquiet.” Emp M
	A need for clarity in communication	“The most difficult thing with dealing with people, is when they are sometimes a bit indirect with their language.” Pro L
		“It is that point of why haven’t they understood what is being asked, or just the processing I think.” Emp K
	Socializing at work can be taxing	“Even one interaction with one person in an hour sometimes can be exhausting.” Pro I
		“They do their best to be very, very sociable, but it seems to obviously be a bit more of a struggle for them than for someone who isn't on the spectrum.” Emp H
	Miscommunications can happen	“I don’t communicate as well as I should with people I kind of assume that people know what I’m talking about, when they have no idea.” Pro G
		“Saying the wrong thing at the wrong time, needing certain information in a way.” Emp C
	Networking is challenging	“People will want to go out for drinks, I usually do because it is networking and you are supposed to, but I find that period really tough.” Pro K
		“There is a lot of ‘let’s sit around and have a drink’, so after a meeting I am a bit ahh! I am peopled out, but I feel like I have to do this so I will.” Pro H
	Mediating the responses of non-autistic colleagues	“I think where the problem occurs is when the non-autistic people can’t relate to the autistic people and then they start shouting and screaming or they start having little bitchy sessions or whatever and then it affects the whole dynamic…But it’s never been the autistic people that have created that.” Emp I
		“Occasionally I will just say something or do something that people find really weird, and I didn’t realise it was weird! And there will be some social misunderstanding that I need to deal with.” Pro J
	Peers in the industry are often scared to make a mistake	“Most casting directors who are concerned about that, are concerned about saying the wrong thing and embarrassing themselves probably… there is definitely a fear of how to speak to people, whether they can speak to people directly, and all sorts of things really.” Emp M
		“People will be basically shitting themselves not quite knowing what to say or do and in practice you just say or do the normal range of things that you normally do but sometimes with a few extra pointers.” Emp A
	The majority of problems can be overcome	“Never anything that was like a major obstacle, never anything that couldn’t be very easily solved.” Emp L
*Not all want to disclose*	Will I be judged negatively?	“Just concerned, that is before someone even meets me, they are going to see me as being needy.” Pro K
		“The real barrier and the real thing I struggle with is just other people’s perceptions, and other people’s misconceptions.” Pro H
	Pigeon-holed to autism-specific work	“Yes, I have Asperger’s Syndrome, but you have to remember, it doesn’t define who I am.” Pro Q
		“I don’t just want it to tie me down to just doing autism related work, or autism related theatre work. There is other stuff I am interested in.” Pro B
	Out and proud	“I’m deliberately quite ‘out’ about it, because I don’t have any problem with it.” Pro I
	Employers aren’t always being told	“I’ve never seen an actor’s profile on Spotlight with a mention of autism. I don’t know if it’s something that is widely documented if an actor does have autism that they put it on their CV as someone with a disability would.” Emp H
		“Actors don’t disclose their disability and it’s not a part of the show so you would never know. So, is that a good thing or a bad thing? I don’t know, but it’s not a visible thing.” Emp B
	A desire to fit in	“It’s a bit infantilising to have to ask for help sometimes.” Emp A
		“They feel they can’t say it because they feel they want to just pass as being normal or they feel like it’s too awkward to ask or they’re embarrassed or something.” Emp B
	Support necessitates disclosure	“They just became more understanding…. I could be honest about the fact that something was a bit loud.” Pro I
		“I’m not shy about coming forward and saying, ‘I’m autistic. This is what I need.’” Pro M
		“In order to ask for help, you have to disclose.” Pro P
*A need for individualised support*	It starts with a conversation	“It’s very much what are you access needs, how can we best support you? Having an open and honest conversation and making sure there’s a system in place.” Emp B
		“It’s about having that frank conversation and seeing how far that frank conversation goes.” Emp F
	Greater understanding can be enough	“The only support I would want is for people to understand why I do certain things and don’t judge me.” Pro N
		“On set no, not extra support as such, just an understanding.” Emp I
	Allowing different modes of working	“We have to make sure that he gets his script a good week in advance, so he's got proper preparation time.” Emp G
		“The director was really aware of that and then would check in with me about light and sound levels in the room and how exercises were going and stuff.” Pro K
		“It might be that hours are different, to avoid packed trains, which is something we’re looking into, because it’s not just about arriving. If you arrive and you’re completely broken because your journey was impossible, then there’s no point being at work.” Emp F
	Support from others	“A mentor would be amazing.” Pro G
		“What you need is somebody to see you, who is not necessarily part of the company, somebody who goes, “Yes, I can see the difficulty you’re having” and maybe you’d talk to.” Pro P
		“We took the decision to employ a chaperone.” Emp N
		“They discuss their support needs together with that person…they might say, you know, “My support needs are this, this and this. I would like you to work with me in this way.” Emp B
		“I feel like internally we have a number of people who are very plugged into being… you know, it’s their job to be up to date.” Emp J
		“I would love it if there was a person to speak to about it.” Emp H
	No time for training	“Well, when are we going to have time to train, attend training?” Emp C
		“The kind of training that has got live people in it is not desired by the film industry, where of course you’ve got a lot of freelance people working so how can you get them because people get together on a project by project basis so how can you all get them in a room at one time? You can’t.” Emp A
		“You spend your life on Google, and training courses!” Emp E
	Support can be inconsistent	“We will find our production in last minute places and often, certainly for the offices and studios, the cheapest places, some of them don’t necessarily have the access requirements or the areas to relax.” Emp N
		“The provision is very very patchy… they will say oh sure we can do that, and then unless you pursue it, nothing actually comes of it.” Pro J
	I will learn when it’s relevant	“If I were to be in a position where I was working regularly with someone with autism then I’d make sure—or if any of my staff were in that position, when they have been in that position, I’ve made sure that they've had training.” Emp D
		“I think online resources, with the best will in the world, people either pay lip service to them or they go looking for them when they need something as opposed to being trained pre-emptively.” Emp N
	A lack of confidence	“I don't feel that confident personally. If the situation arose, where without time to prepare, without time to receive any awareness training, where I was required to work with work extensively with someone with autism I'd probably be quite uncomfortable with that.” Emp D
		“I think a lot of what, me and casting directors would find daunting about learning more about this, is not wanting to get anything wrong, being able to examine your own insecurities, hesitations.” Emp R
	Unaware of resources	“I do not know where to point any autistic employee if she felt or he felt that they needed more help.” Emp F
		“I wouldn't be able to point someone else in the direction of information outside of the organisation. I don’t know if they're entitled to any kind of right to work support.” Emp D
	The burden of advocacy	“I do wish they knew more, because you spend a lot of time and a lot of energy having to explain yourself, and that’s really hard. Everybody else doesn’t have to do that.” Pro I
		“Most of the time, people just don’t really know what it means, they don’t really know what to do with the information because they don’t really know how [autism] affects you.” Pro R
	An openness to learn more	“I would like to know more about that and also if there’s a way of engaging with autistic actors certainly a lot more than I have done.” Emp H
		“It would be wonderful to know how I can make my rehearsal process and my rehearsal rooms and my auditions and meeting actors and things more accessible so that I can meet the best people working whether they are autistic or not.” Emp L

### Autism can Bring Strengths

The autistic professionals were keen to emphasise that although being autistic could be associated with some challenges in the workplace, their autistic characteristics were also a source of strength, and that some of their traits seemed particularly well-suited to their performing arts work. For example, some professionals described being *scholars of human expression*: “I had to learn to understand body language, behaviours, facial expressions that sort of thing. I had to study them and from doing that, I think it’s made me into a better actor” [Pro R]. Many discussed their *high engagement with their work*, and how they could become engrossed with a particular task and maintain focus for longer than their non-autistic colleagues: “Hyper-focus is a bit beneficial for that, so I get really involved – yes, so long hours don’t particularly bother me” [Pro J]. Often, *a detail-oriented approach* accompanied this high level of focus: “I possibly just generally pay a bit more attention to the detail than other people might” [Pro L]. These traits were also noticed by employers: “There are characteristics of autism that seem really great for this kind of work, which is the attention to detail, and the determination to get things exactly right, which is brilliant” [Emp M]. Both groups felt that viewing the world through an autistic lens led to a unique perspective. One professional explained: “I think Asperger’s lends itself to creative thinking that you *see the world a bit differently*, and that is actually quite a useful talent for the arts!” [Pro J]. This sentiment was echoed by employers: “Autistic people in acting, there’s a boldness in trying things” [Emp A].

### A Challenging Profession

Both professionals and employers identified several aspects of performing arts work that could be challenging for autistic professionals. Many professionals spoke of sometimes finding that *the workplace can be overwhelming*: “I will be the person that’s likely to meltdown and loses it because I can’t hold it in, or I get too stressed” [Pro I]. The employers acknowledged how autistic professionals might find the sensory environment at work particularly challenging: “The lighting in some parts of the building can be a real barrier to autistic people. Sometimes, unfortunately, that’s the only space that we can do certain things” [Emp F].

Professionals also described how *auditions can cause extreme anxiety*: “Auditions I hate. I do not do well with auditions” [Pro O]. Employers recognised that autistic professionals may *struggle adapting to last-minutes changes* involved in a lot of performing arts work: “I understand that last-minute changes to arrangements, which I am afraid does happen in this industry, can actually cause a bit of disquiet” [Emp M]. Professionals confirmed this: “Things do change last minute, and I find that really difficult, I find that really stressful internally” [Pro K]. Some professionals struggled with understanding instructions from colleagues or other staff and had *a need for clarity in communication*: “The most difficult thing with dealing with people, is when they are sometimes a bit indirect with their language” [Pro L]. Employers also picked up on these difficulties: “It is that point of why haven’t they understood what is being asked, or just the processing I think” [Emp K].

Both employers and professionals recognised that a great deal of challenges for autistic performing arts professionals were often rooted in their social interactions with others. Professionals spoke of how occasional *miscommunications can happen*: “I don’t communicate as well as I should with people. I kind of assume that people know what I’m talking about, when they have no idea” [Pro G] and how the expectation of *socialising at work can be taxing*: “Even one interaction with one person in an hour sometimes can be exhausting” [Pro I]. Some employers also noticed these difficulties in the workplace: “They do their best to be very, very sociable, but it seems to obviously be a bit more of a struggle for them than for someone who isn’t on the spectrum” [Emp H]. Many professionals described difficulties with navigating the expectation that they would also socialise with colleagues outside of work to network, and how managing energy levels for *networking is challenging*: “People will want to go out for drinks. I usually do because it is networking and you are supposed to, but I find that period really tough” [Pro K].

Some of the employers who had experience working with autistic employees described one unanticipated challenge around *mediating the responses of non-autistic colleagues* to their autistic employee(s): “I think where the problem occurs is when the non-autistic people can’t relate to the autistic people and then they [non-autistic people] start shouting and screaming or they start having little bitchy sessions or whatever and then it affects the whole dynamic. But it’s never been the autistic people that have created that” [Emp I]. Alongside having to manage the responses of colleagues already working alongside autistic employees, employers also commented that their *peers in the industry are often scared to make mistakes* and seem apprehensive about the prospect of working with autistic people: “Most casting directors who are concerned about that, are concerned about saying the wrong thing and embarrassing themselves probably… there is definitely a fear of how to speak to people, whether they can speak to people directly, and all sorts of things really” [Emp M]. While acknowledging that there were challenges when working with autistic employees, employers were also keen to emphasise that *the majority of problems can be overcome*: “[There was] never anything that was like a major obstacle, that couldn’t be very easily solved” [Emp L].

### Not All Want to Disclose

Many professionals reflected on whether disclosing being autistic to their employers or colleagues would bring more advantages or disadvantages. In particular, some worried about whether they would *be judged negatively* by their colleagues or employers if they revealed that they were autistic: “The real barrier and the real thing I struggle with is just other people’s perceptions, and other people’s misconceptions” [Pro H]. Some professionals were also concerned that if they revealed they were autistic to potential employers or their agents, this disclosure would place them at risk of being *pigeon-holed into autism-specific work* and perhaps limit their hiring opportunities: “I don’t just want it to tie me down to just doing autism-related work, or autism-related theatre work. There is other stuff I am interested in” [Pro B]. In line with this view, many employers spoke of not being aware of whether they had encountered anyone autistic and *not being told about diagnoses*: “I’ve never seen an actor’s [online casting] profile with a mention of autism. I don’t know if it’s something that is widely documented if an actor does have autism that they put it on their CV as someone with a disability would” [Emp H]. They suggested that this was probably due to people not disclosing their autism rather than the possibility that they were not working with anyone autistic. Some employers reflected on why people may not be disclosing and surmised that this may be due to *a desire to fit in* with non-autistic colleagues and be considered in the same light as other members of staff: “They feel they can’t say it because they feel they want to just pass as being normal or they feel like it’s too awkward to ask or they’re embarrassed or something” [Emp B]. Conversely, there were a minority of professionals who were happy to be consistently ‘*out*’ about being autistic: “I’m deliberately quite ‘out’ about it, because I don’t have any problem with it” [Pro I]. Many professionals recognised that in order *to receive support it was often necessary to disclose* their diagnosis to their employer: “I’m not shy about coming forward and saying, “I’m autistic. This is what I need” [Pro M], and a few found that this not only led to workplace accommodations but also colleagues who recognised and were more empathetic to their needs: “They just became more understanding. I could be honest about the fact that something was a bit loud” [Pro I].

### A Need for Individualised Support

Employers recognised that every autistic person with whom they may work will have a unique set of characteristics and needs, and therefore described the individualised approach that they had previously provided or could potentially offer. The majority, however, were not fully confident about what this might look like in practice. Some advocated for *starting with a conversation* with the individual who may need support: “It’s about having that frank conversation and seeing how far that frank conversation goes” [Emp F]. They also suggested that in some cases all that is required in the way of support is *greater understanding*, consideration and awareness: “On set no, not extra support as such, just an understanding” [Emp I]. This sentiment was echoed by professionals: “The only support I would want is for people to understand why I do certain things and don’t judge me” [Pro N]. Some employers spoke of *allowing different modes of working* for employees who needed it, from providing quiet spaces for employees, to ensuring that autistic actors received their scripts a week in advance of shooting to allow proper preparation time, and allowing them come into work at different times where possible: “It might be that hours are different, to avoid packed trains, which is something we’re looking into, because it’s not just about arriving. If you arrive and you’re completely broken because your journey was impossible, then there’s no point being at work” [Emp F].

Both employers and professionals spoke about the varied ways that *support from others* could be useful. Some employers were able to offer their autistic employees onsite support from a support-worker. This meant that there was someone for the autistic employees to speak directly with about any issues they were having, and also, who could advocate for their needs to the rest of the company: “They discuss their support needs together with that person. They might say, you know, ‘My support needs are this, this and this. I would like you to work with me in this way’” [Emp B]. When professionals were asked about the support they’d like to see more of, their answers often centred around wanting someone to consult, such as a mentor, about any work-based difficulties and how to progress their careers: “What you need is somebody to see you, who is not necessarily part of the company, somebody who goes, ‘Yes, I can see the difficulty you’re having’ and maybe you’d talk to” [Pro P]. For some professionals, it was important that a potential mentor should also be autistic. Similarly, many employers also spoke about their desire to consult with someone who could support them with making disability accommodations within their company. Some employers felt that they already had someone in a role within their organisation who could respond to the potential support needs of an autistic employee: “I feel like internally we have a number of people who are very plugged into being… you know, it’s their job to be up to date” [Emp J], while others expressed a desire for such a person: “I would love it if there was a person to speak to about it” [Emp H]. What was common across employers, however, was that there was a tendency to rely upon someone else within the workplace to have the requisite knowledge and to implement that support, rather than be that person themselves.

In contrast to the examples of support given by the employers in our study, the majority of the autistic professionals felt that the employers they had encountered across their careers did not have adequate knowledge about autism and considered what that meant for them in the context of the workplace. They communicated their fatigue over the *burden of advocating* for themselves: “I do wish they knew more, because you spend a lot of time and a lot of energy having to explain yourself, and that’s really hard. Everybody else doesn’t have to do that” [Pro I]. Many employers were aware that they could improve their knowledge of autism and did speak of *an openness to learning more* and a desire to improve: “It would be wonderful to know how I can make my rehearsal process and my rehearsal rooms and my auditions and meeting actors and things more accessible so that I can meet the best people working” [Emp L]. Some of the employers, however, were *not confident* that with their current levels of knowledge they could presently provide adequate support: “I don’t feel that confident personally. If the situation arose, where without time to prepare, without time to receive any awareness training, where I was required to work extensively with someone with autism I’d probably be quite uncomfortable with that” [Emp D]. The majority of employers were *unaware of any resources* available to autistic professionals outside of what their own organisations could provide: “I do not know where to point any autistic employee if she felt or he felt that they needed more help” [Emp F]. Some of the employers were keen to provide appropriate support for autistic employees if, and when, they had them. Rather than pre-emptive training, some employers wanted to respond as and *when they felt it was relevant*: “If I were to be in a position where I was working regularly with someone with autism then I’d make sure – or if any of my staff were in that position, when they have been in that position – I’ve made sure that they've had training” [Emp D]. While many employers indicated a desire to learn more about supporting autistic members of staff, they emphasised that educational resources needed to be easily accessible and not overly time-consuming. This was due to the *time commitment involved in attending training*, the coordination, and the financial constraints of potentially paying to train not only themselves, but large numbers of staff, who may be only working for the company for short periods of time: “The kind of training that has got live people in it is not desired by the film industry, where of course you’ve got a lot of freelance people working so how can you get them because people get together on a project by project basis so how can you all get them in a room at one time? You can’t” [Emp A]. Alongside, the impermanence of staff in their employ, the location of where work would take place could change as well. With changing locations, employers commented on the difficulty of maintaining access requirements: “We will find our production in last-minute places and often, certainly for the offices and studios, the cheapest places, some of them don’t necessarily have the access requirements or the areas to relax” [Emp N]. Some professionals expressed their frustration at how promised *support could be inconsistently implemented*, which could suggest a lack of recognition from employers of how vital the consistency of support can be to some autistic professionals: “The provision is very, very patchy… they will say ‘oh sure we can do that’, and then unless you pursue it, nothing actually comes of it” [Pro J].

## Discussion

This study examined the employment experiences of autistic performing arts professionals and performing arts employers in the UK. Importantly, members from both groups recognised key areas of challenge for autistic professionals and also the strengths that autistic professionals can bring to this field. Many autistic professionals reported that there was inadequate employment-based support, and these claims were corroborated by the low levels of knowledge that many of the employers themselves reported regarding autism and appropriate methods of support. These findings support the key claims within the adapted OIMIB framework (Annabi and Locke 2019) of the critical relationship between the levels of knowledge that neurotypical colleagues or employers have around autism and the amount of barriers that autistic people face in employment.

The autistic professionals and the employers were keen to emphasise how autistic characteristics can be advantageous in the workplace with both groups highlighting autistic strengths, such as paying attention to detail, notably high levels of focus, and taking often uniquely creative approaches to tasks. These are traits that have been recognised as skills that autistic people can bring to the workplace (Baron-Cohen et al. 2009; de Schipper et al. 2016; Hagner and Cooney 2005; Ham et al. 2014; Scott et al. 2018). One talent that proved particularly useful for autistic performers was the reported encyclopaedic knowledge of facial expressions and body language that they had built up from studying other people over the course of their lives. This phenomenon reflects findings that autistic people process faces and emotional expressions in a more extrinsic way than neurotypical people (Harms et al. 2010) and often use masking or compensatory strategies for “putting on my best normal” (Hull et al. 2017), which the professionals were able to harness for their performance work.

Autistic professionals and employers also recognised, however, how challenging working in the performing arts can be for autistic people. Many of the challenges identified by both groups centred on aspects of social communication and interaction. The social challenges may be exacerbated in this particular industry, in which workers are often employed on a project-based system, which means professionals are constantly having to seek new employment and undergo numerous auditions/interviews to maintain employment (Menger 2006). This may be particularly burdensome to autistic professionals as they report high anxiety around auditions and may struggle with aspects of job interviews such as small talk (VanBergeijk et al. 2008). Like our autistic participants, others have reported that social and collegial relationships at work can be one of the most challenging aspects of work (Baldwin et al. 2014). Many of the employers interviewed herein also remarked on this issue. Previous research has found social difficulties with colleagues and supervisors can hinder job performance and can even lead to job termination (Bolman 2008; Hendricks 2010; Hurlbutt and Chalmers 2002, 2004).

Alongside the difficulties with social communication and interaction, many of the autistic professionals spoke of feeling anxious generally when at work, and some described feeling extreme anxiety in response to auditions. These findings reflect existing research showing the high prevalence of co-occurring mental health conditions (especially anxiety) in autistic people (Lever and Geurts 2016; Simonoff et al. 2008; Strang et al. 2012) and the high levels of stress and anxiety that autistic people report in the workplace (Hurlbutt and Chalmers 2004). It also echoes our own research, which found that performing arts professionals with elevated autistic traits are more likely to report clinically-significant levels of mental health issues (Buckley et al. 2020). Many autistic people can be hypersensitive to certain everyday sensory stimuli, such as light and sound (Marco et al. 2011), which can make being in certain environments challenging. Struggling to cope with sensory stimuli in the workplace can cause anxiety, alongside difficulties with social communication and interactions, unpredictable situations, or when last-minute changes occur (Burt et al. 1991; Hurlbutt and Chalmers 2004; Remington and Pellicano 2018). Again, these issues may be intensified within the performing arts workplace. The physical setting can often change both within and between jobs, such as filming in a variety of locations for a project, which means that access requirements may not always be consistently met for autistic employees, such as sensory needs. Employees are typically expected to be able swiftly to adapt to new working environments, which can also present challenges for autistic people (Dipeolu et al. 2015), who often have difficulties with executive function, especially cognitive flexibility (Powell et al. 2017; Wallace et al. 2016).

One less anticipated challenge for some of the employers was having to mediate the negative responses of non-autistic colleagues to their autistic employee(s). These negative attitudes displayed by colleagues towards their autistic co-workers are highlighted as one of the barriers to successful employment described in the adapted OIMIB framework (Annabi and Locke 2019). Neurotypical adults can struggle to interpret correctly the behaviour (Sheppard et al. 2016) and facial expressions (Brewer et al. 2016) of autistic people, and are more reluctant to interact with them compared to other neurotypical people, based on first impressions (Sasson et al. 2017). This behaviour noticed by employers is reflective of Milton’s (2012) ‘double empathy’ problem, where neurotypical people do not behave in an empathetic way towards autistic people, as they would to neurotypical others.

Alongside identifying the many challenges they face in the workplace, the autistic professionals also provided many suggestions for how they could be supported to overcome these challenges. For instance, the professionals suggested support in the form of assistance with social situations, access to quiet spaces at work, and for some, simply for colleagues to have a greater understanding of autism and tolerance of behavioural differences. Employers can offer adjustments in management style such as ensuring instructions are precise, that information is communicated in a way that suits the individual employee, and by creating a work environment where people feel comfortable to opt in or out of socialisation with minimal consequence. The autistic professionals spoke about the expectation and importance of networking as a way to further their careers, which is acknowledged as a crucial aspect of career progression in the arts (Bennett 2009), and how challenging this could be. One way that autistic professionals could be supported with networking is through mentorship from those with more established careers and experience in the industry, and this is a type of support professionals in this study identified as desirable. Mentoring can be an effective form of support for autistic people in employment (Dawkins et al. 2016; Dipeolu et al. 2015; Nicholas et al. 2018), although the feasibility and effectiveness of mentoring has never been specifically tested for those working in the performing arts field. The performing arts professionals in this study wanted mentors who could offer guidance on career progression, give feedback on applications for grants, and recognise and help them manage workplace difficulties.

The autistic professionals were able to pinpoint precisely the support they needed. Yet, the vast majority of them reported that they had received little, if any, of this support, and when it was received it was inconsistent across different workplaces. Our previous work showed that performing arts professionals with high levels of autistic traits are more likely than those with low levels of autistic traits to need and want more support in relation to their work (Buckley et al. 2020). Other studies outside the field of the performing arts have also demonstrated that the majority of autistic workers report receiving no workplace adjustments to support them (Baldwin et al. 2014; Beardon and Edmonds 2007; López and Keenan 2014), despite wanting to receive such support in their current and future workplaces (Baldwin et al. 2014). For those that had received some support, it had been both difficult to find or inconsistently implemented. This finding is consistent with research that suggests that even when workplaces adopt formal disability policies, these are often not followed by changes in practice (Hoque and Noon 2004).

One plausible reason why professionals reported a lack of support in their workplaces may be due to not disclosing their diagnoses, and with autism often being a hidden disability, employers may not realise that they have autistic employees who require support (Johnson and Joshi 2016; Sarrett 2017). There were several reasons for autistic professionals’ apprehension around disclosure, primarily the concern that non-autistic colleagues would judge them negatively if they found out they were autistic, which is a belief held by many autistic people (Davidson 2010; Davidson and Henderson 2010; Hull et al. 2017). Research suggests that it is common for disabled people to be judged as less capable in a work context than non-disabled people (Colella and Varma 1999), and autistic people experience more immediate, negative reactions towards them than non-autistic others (Sasson et al. 2017). The potential negative reactions of non-autistic colleagues and employers to disclosure of autism is a justified concern for autistic people.

Some of the autistic performing arts professionals had had positive experiences with disclosure and found colleagues had become more understanding of their differences post-disclosure. This finding supports previous research showing that when an autism diagnosis is disclosed, neurotypical people form first impressions and perceive behaviours more positively than when an autism diagnosis is not disclosed (Brosnan and Mills 2016; Sasson and Morrison 2019). Some of the autistic professionals emphasised that they saw disclosure as a necessary step to gain support, which is a sentiment echoed by other autistic people (Huws and Jones 2008). Non-disclosure may be one key barrier to support. One way in which autistic professionals could be supported with this is through guidance, mentorship, and legal advice regarding disclosure to employers. Ideally this support would be developed, co-produced, and evaluated for its effectiveness in collaboration with autistic people to ensure that resulting research and practice will be relevant and specific to their needs (Fletcher-Watson et al. 2019; Milton et al. 2017).

The employers had widely varying experience regarding working with autistic people, from some having never knowingly worked with an autistic person, to others who work with autistic performing arts professionals on a regular basis. Nevertheless, the majority of employers revealed that they did not feel as if they knew enough about working with autistic people or the ways in which autistic employees could be supported, both internally and externally to their workplaces. This finding reflects previous research that has found that employers do not possess a great deal of information about working with disabled people (Rashid et al. 2017) and a lack of autism knowledge is highlighted as another key barrier to successful employment in the adapted OIMIB framework (Annabi and Locke 2019).

Many of the employers were dependent on others for knowledge on what support might be needed and how it would be implemented. This lack of confidence and desire to use external support is consistent with previous research, which has found that employers are reluctant to provide workplace support without the guidance of disability employment organizations (Howlin et al. 2005), and that employers feel that they need to rely on external support to best support their autistic employees (Scott et al. 2015).

Employers had mixed views regarding what type of resources would be appropriate to help them learn more about supporting autistic people in the workplace. Some cited the lack of time they felt they had to attend training courses and wanted resources they could engage with when they needed to, such as an online repository of information. Others wanted a specially trained consultant to talk with and to be able to ask questions to someone as they came up. Providing a safe and non-judgmental environment for employers to learn more seems to be an important aspect of how we can improve knowledge, support, and perhaps even increased employment for autistic people. Employers will benefit from resources and training that are informative, practical, and can be easily accessed and implemented (Rashid et al. 2017; Unger and Kregel 2003). It will also be important to co-design and co-produce any support with both employers and autistic professionals in the performing arts to ensure that training and resources are relevant to their workplaces and tailored to people’s individual needs.

### Limitations

This research is not without its limitations. First, although the sample was geographically diverse, the current work nevertheless examined the experiences of a selective sample of autistic performing arts professionals and performing arts employers. Nearly all of the professionals that were included in this study identified publicly as autistic to some degree, if not specifically at work. We did not specifically recruit employers who had worked previously with disabled employees. Nonetheless, the performing arts employers in this study seemed to be open to considering disability support in the workplace and the supported employment of autistic people, in particular, although we cannot be sure that these views represent those of all such employers. The employers reported that they were keen to learn more and improve their knowledge and ensure support for autistic employees was sufficient. Although this is a heartening response, we must be cautious that these statements are not simply the result of social desirability bias (Grimm 2010). Nevertheless, respondents did not only report positive aspects of working with autistic people, many were willing to discuss their challenges and concerns as well. Second, one of the autistic performing arts professionals self-diagnosed as autistic but did not yet have a clinical diagnosis of autism. That this professional’s answers were similar in nature to the professionals with existing diagnoses warrants confidence in our decision to retain them in the sample.

## Conclusion

In conclusion, our findings are novel in that they provide the first understanding of the experiences of autistic performing arts professionals in the UK and the attitudes and support offered by UK-based performing arts employers to autistic employees. The autistic performing arts professionals reported an overall lack of support from their employers. They suggested that they could be supported through mentorship, greater accessibility and support in the workplace, and increased understanding and acceptance of autism from their colleagues and employers. The performing arts employers, whilst demonstrating open attitudes towards employing and supporting autistic people, did not unanimously feel confident in being able to currently provide that support. Future research is needed to test the feasibility and effectiveness of types of support for autistic performing arts professionals, especially mentor-based programmes, alongside improving the knowledge and confidence of performing arts employers.

## Footnotes

1 We included one individual who self-identified as autistic but had not yet received a formal diagnosis. Their inclusion was important because there are lengthy waiting lists for adult diagnostic services in the UK (Unigwe et al. 2017), and many older autistic adults, including autistic women and non-binary people may have been mis-diagnosed or missed out on a diagnosis altogether (Gould 2017; Kirkovski et al. 2013; Lai and Baron-Cohen 2015; Linton et al. 2014).
